# Deletion of human metapneumovirus M2-2 increases mutation frequency and attenuates growth in hamsters

**DOI:** 10.1186/1743-422X-5-69

**Published:** 2008-06-03

**Authors:** Jeanne H Schickli, Jasmine Kaur, Mia MacPhail, Jeanne M Guzzetta, Richard R Spaete, Roderick S Tang

**Affiliations:** 1 Research Dept, MedImmune, Mountain View, CA 94043, USA

## Abstract

**Background:**

Human metapneumovirus (hMPV) infection can cause acute lower respiratory tract illness in infants, the immunocompromised, and the elderly. Currently there are no licensed preventative measures for hMPV infections. Using a variant of hMPV/NL/1/00 that does not require trypsin supplementation for growth in tissue culture, we deleted the M2-2 gene and evaluated the replication of rhMPV/ΔM2-2 virus *in vitro *and *in vivo*.

**Results:**

*In vitro *studies showed that the ablation of M2-2 increased the propensity for insertion of U nucleotides in poly-U tracts of the genomic RNA. In addition, viral transcription was up-regulated although the level of genomic RNA remained comparable to rhMPV. Thus, deletion of M2-2 alters the ratio between hMPV genome copies and transcripts. *In vivo*, rhMPV/ΔM2-2 was attenuated compared to rhMPV in the lungs and nasal turbinates of hamsters. Hamsters immunized with one dose of rhMPV/ΔM2-2 were protected from challenge with 10^6 ^PFU of wild type (*wt) *hMPV/NL/1/00.

**Conclusion:**

Our results suggest that hMPV M2-2 alters regulation of transcription and influences the fidelity of the polymerase complex during viral genome replication. In the hamster model, rhMPVΔM2-2 is attenuated and protective suggesting that deletion of M2-2 may result in a potential live vaccine candidate. A more thorough knowledge of the hMPV polymerase complex and the role of M2-2 during hMPV replication are being studied as we develop a potential live hMPV vaccine candidate that lacks M2-2 expression.

## Background

Human metapneumovirus (hMPV) infection can cause acute respiratory illness in young infants, the immunocompromised, and the elderly [1-3]. HMPV infection has been detected in 4 to 15% of pediatric patients hospitalized with acute lower respiratory infections [4-10]. Currently there are no licensed measures to prevent hMPV disease.

Based on analyses of genomic sequences hMPV has been assigned to the metapneumovirus genus of the pneumovirus subfamily within the paramyxovirus family [11,12]. The genome contains 8 transcription units with at least 9 open reading frames (ORFs) that encode a nucleocapsid protein (N), a matrix protein (M), a phosphoprotein (P) that likely associates with the polymerase complex, a fusion glycoprotein (F), an attachment glycoprotein (G), a large polymerase protein (L), a small hydrophobic protein (SH), and two proteins (M2-1 and M2-2) encoded by overlapping ORFs in the M2 gene. Among paramyxoviruses, SH is found in rubulaviruses and pneumoviruses, while M2 is found only in pneumoviruses. The functions of M2 proteins have not been studied extensively.

Mutants of hMPV have been constructed by deleting M2-1, M2-2, SH or G, either individually or in combination, using the CAN97-83 isolate of hMPV, which requires trypsin for growth in cell culture [13,14]. Recombinant hMPV lacking either M2-2 or G were attenuated and immunogenic in African green monkeys and have been proposed as promising vaccine candidates [15]. Such a suitably attenuated live hMPV is desirable because it would deliver the nearly complete set of viral antigens and closely mimic a natural hMPV infection.

To construct a rhMPVΔM2-2 virus that can replicate efficiently in Vero cells without trypsin supplementation, we engineered the M2-2 deletion in a different subtype A hMPV strain. This recombinant strain is derived from hMPV/NL/1/00, and contains F_2_/F_1 _cleavage-enhancing mutations in the F gene, a property that could facilitate the testing and manufacture of potential live hMPV vaccine candidates [16,17]. The impact of the physical deletion of M2-2 on hMPV replication, and genetic stability in tissue culture were evaluated. rhMPV/ΔM2-2 exhibited somewhat restricted replication in Vero cells, but was significantly attenuated in hamsters. Hamsters immunized with rhMPV/ΔM2-2 were protected from experimental challenge with *wt*hMPV/NL/1/00. The deletion of M2-2 resulted in higher levels of viral mRNA transcripts in tissue culture, giving rise to aberrant ratios of genomic RNA to viral transcripts. In addition, previously unreported genetic instability was observed, resulting in a higher frequency of point mutations and random insertions of U nucleotides in poly-U tracts of the rhMPV/ΔM2-2 genomic RNA.

## Results

### Expression of M2-2 is not required for hMPV replication in Vero cells

**Figure 1:**
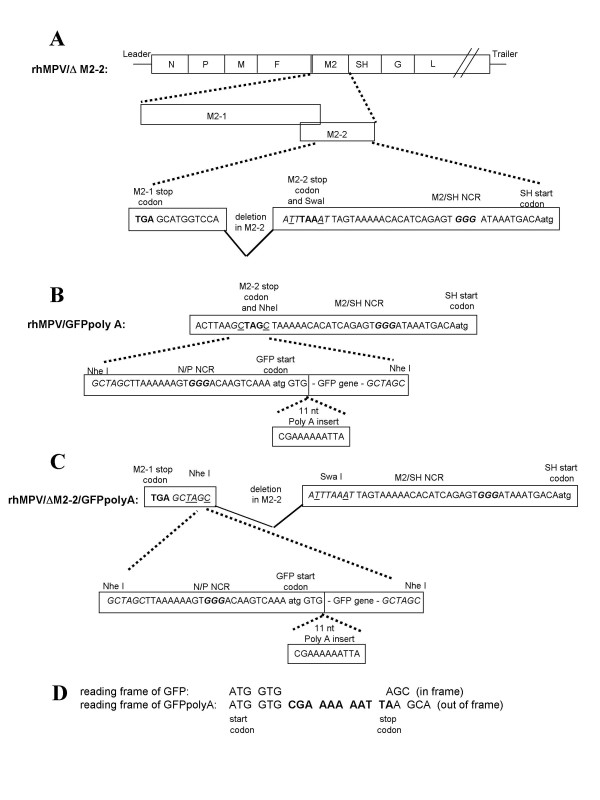
**Construction of cDNA for rhMPV/ΔM2-2, rhMPV/GFPpolyA and rhMPV/ΔM2-2/GFPpolyA**. A) rhMPV/ΔM2-2 has a deletion in the M2-2 gene adjacent to a SwaI site. Nucleotides that were modified to introduce the SwaI site are underlined. Translational stop codons are bold and the intergenic (IG) sequence is bold italics. B) To construct rhMPV/GFPpolyA, an NheI site was introduced at the M2-2 stop codon of rhMPV and an NheI-N/P-GFP-polyA-NheI cassette was inserted. The modified nucleotides are underlined, the stop codon is bold and the IG sequence is bold italics. C) To construct rhMPV/ΔM2-2/GFPpolyA, an NheI site was introduced between the stop codon of M2-1 (bold) and the SwaI site (italics) in rhMPV/ΔM2-2 and an NheI-N/P-GFP-polyA-NheI cassette was inserted. The modified nucleotides are underlined and the IG sequence is in bold italics. D) The reading frame of GFP is aligned with that of GFPpolyA to show the stop codon and frame shift resulting from the 11 nt insertion.

Recombinant hMPV harboring a deletion in M2-2 gene was recovered from rhMPV/ΔM2-2 cDNA. The M2-2 deletion was designed to preserve the native ORF of M2-1, which overlaps the M2-2 ORF by 51 nucleotides. The first 21 amino acids of the putative M2-2 protein and the entire M2/SH non-coding region (NCR) were maintained (Figure 1A). Recombinant rhMPV/ΔM2-2 was efficiently recovered. RT-PCR was performed on the recovered rhMVP/ΔM2-2 virus to confirm the presence of the M2-2 deletion.

**Figure 2:**
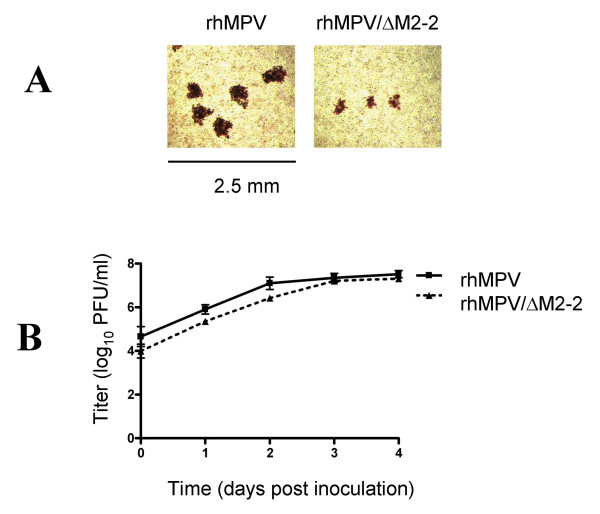
**Growth of rhMPV and rhMPV/ΔM2 in Vero cells**. A) Vero cell monolayers were inoculated with rhMPV or rhMPV/ΔM2-2 and incubated at 35°C under 1% methylcellulose in optiMEM. At 6 days p.i., the cells were fixed in methanol and immunostained with ferret polyclonal antibody directed to hMPV followed by anti-ferret horse radish peroxidase-conjugated antibody. The immunostained plaques were treated with 3-amino-9-ethylcarbazole for visualization. B) Replicate cultures of Vero cells were inoculated with rhMPV or rhMPV/ΔM2-2 at MOI of 0.1 PFU/cell and incubated at 35°C. Supernatants and cells were harvested daily for 4 days. Titers were determined by plaque assay in Vero cells. The graph represents an average +/- SD titer of three independently performed experiments.

In Vero cells, rhMPV/ΔM2-2 plaques were less than 50% the size of rhMPV plaques (Figure 2A). A 4-day multi-cycle growth curve was performed in Vero cells, a cell-line used for production of live vaccines, to compare the replication kinetics of rhMPV/ΔM2-2 and rhMPV. Data for the replication curves of these viruses were collected from three independently performed infections. The peak titer of rhMPV/ΔM2-2 in Vero cells was 7.22 +/- 0.16 log_10 _PFU/ml, which was not significantly lower than the 7.52 +/- 0.29 log_10 _PFU/ml titer achieved by rhMPV (Figure 2B). However, the plaque size of rhMPV/ΔM2-2 was markedly diminished compared to rhMPV. Thus, hMPV M2-2 is dispensable for replication in Vero cells.

### Sequences of rhMPV/ΔM2-2 contain major subpopulations with mutations and insertions of A nucleotides

**Figure 3:**
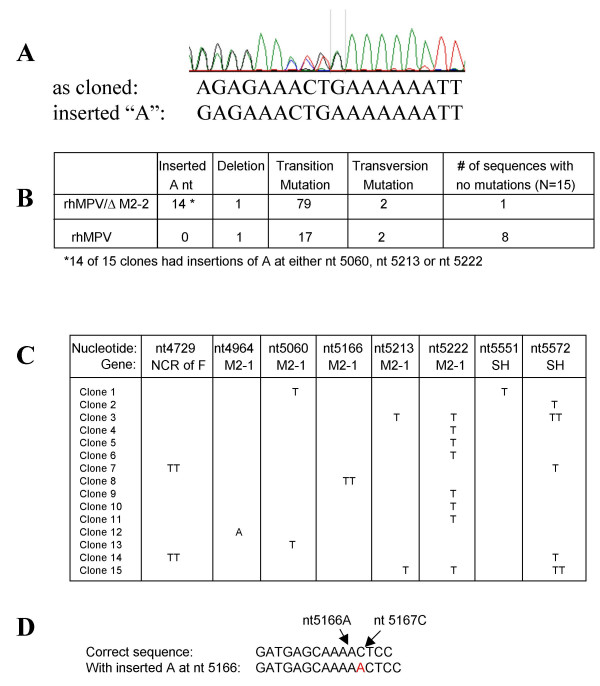
**Chromatogram and frequency of A insertions and point mutations in rhMPV/ΔM2-2 compared to rhMPV**. A) A chromatogram of the RT-PCR product derived from P4 of rhMPV/ΔM2-2, spanning nt4536 in F to nt6205 in NCR of SH, contained this sequence showing two subpopulations. One population is the correctly cloned sequence; the second population has one inserted A nt (sense direction) at nt5222 in the M2-1 gene. B) To assess the relative frequency of mutations, RT-PCR fragments spanning nt4536 in F to nt5623 in SH were obtained from rhMPV/ΔM2-2 or rhMPV using one-step RT-PCR, and were cloned into pCR2.1 plasmids. Among 15 independent plasmids the number of inserted As, single nt deletions, and point mutations (transition or transversion) for each virus were tabulated. 14 of the 15 (93%) rhMPV/ΔM2-2RT-PCR products had an inserted A (sense direction) nucleotide. No fragments containing A nucleotide insertions were detected in any of the 15 RT-PCR fragments spanning the identical region in P4 of rhMPV. C) To study frequency of mutations in genomic RNA, RT-PCR fragments spanning nt4536 to nt5623 were obtained from rhMPV/ΔM2-2 using two-step RT-PCR, and were cloned into pCR2.1 plasmids. Nucleotide insertions were predominantly T (genomic antisense direction), with one A, and were distributed among 8 locations in the fragments. D) To describe the position where insertion of an A was observed, the nt number of the last A in the poly A tract is used, though it is not known which A residue in the poly A tract is the inserted residue. The example shown is A inserted at nt5166.

During the preparation of viral stocks, we noted several mutations in rhMPV/ΔM2-2. To further assess the genetic stability of rhMPV/ΔM2-2, one-step RT-PCR was performed on a virus stock that was serially passaged 4 times in Vero cells. Sequence analysis of an RT-PCR product spanning the M2 and SH genes (nt4536 to nt6205) revealed nucleotide polymorphisms in several poly A tracts (sense direction) in the M2-1 and SH genes. Figure 3A shows a representative chromatogram of the sequence of an RT-PCR product generated from a rhMPV/ΔM2-2 virus stock. The wild-type sequence AGAGAAACTGA_6_TT is shown overlapping another sequence containing an inserted A in the poly A_6 _tract. Three independently derived virus stocks of rhMPV/ΔM2-2 had major subpopulations with inserted A nucleotides (nts) at nt5060, nt5166 or nt5222 in M2-1, each of which would cause a premature translation termination in the M2-1 ORF. (See figure 3D for numbering of A insertions). Subpopulations with inserted A's were also detected at nt5551 or nt5572 in SH that would result in premature translation termination in the SH ORF.

To compare the frequency of inserted A nucleotides in rhMPV/ΔM2-2 to that in rhMPV, RT-PCR products spanning nt4536 in F to nt5623 in SH were obtained from a passage 4 virus stock of rhMPV/ΔM2-2 or rhMPV. For this study, both positive sense and negative sense RNA were amplified using a one-step RT-PCR reaction. 1 kb RT-PCR fragments were inserted into pCR2.1 plasmids and 15 independent plasmids were sequenced. Surprisingly, 14 of the 15 (93%) cloned RT-PCR products of rhMPV/ΔM2-2 had an inserted A nucleotide at nt5060, nt5213 or nt5222 in the M2-1 gene (Figure 3B): there were 6 clones with insertion of A at nt5060, 2 with insertion at nt5213 and 6 with insertion at nt5222. Insertions of U, C or G were not observed. In sharp contrast, no A nucleotide insertions were detected in 15 cloned RT-PCR products derived from the identical region of rhMPV. Transitions, transversions, and deletions were also observed for rhMPV/ΔM2-2 in addition to insertions of A. For rhMPV/ΔM2-2, 14 of 15 cloned RT-PCR sequences exhibited a total of 79 transition mutations, 2 transversions, and 1 deletion. Only 1 cloned sequence from rhMPV/ΔM2-2 had no nucleotide changes. In comparison, 7 of 15 cloned RT-PCR products of rhMPV showed a total of 17 transitions, 2 transversions, and 1 deletion. Eight cloned RT-PCR sequences from rhMPV had no nucleotide changes (Figure 3B). Thus, by passage 4, both rhMPV and rhMPV/ΔM2-2 contained heterogeneous subpopulations and rhMPV/ΔM2-2 had a higher frequency of transition mutations and a propensity for insertion of A nucleotides in poly A tracts, compared to rhMPV.

To determine whether U insertions were present in the antisense genome, a two step RT-PCR was performed to specifically amplify only genomic RNA. Again, total RNA was extracted from a passage 4 stock of rhMPV/ΔM2-2 and the region from nt4536 in F to nt5623 in SH was amplified. 1 kb RT-PCR products were inserted in pCR2.1 and 15 individual plasmids were sequenced. All 15 cloned RT-PCR products contained an insertion of 1 or 2 T nts (antisense) in either the F gene (non-coding region), the M2-1 gene or the SH gene (Figure 3C). One sequence had an A inserted at nt4964 in the M2-1 gene. However, no insertions of C or G were observed. The 15 cloned RT-PCR products also contained 18 transitions and 4 transversions. Thus, there is a high frequency of U insertions in the genomic RNA suggesting that insertions were propagated in the viral genome. Whether the insertion events occurred during synthesis of the genomic or antigenomic RNA cannot be determined from these data.

We next examined the frequency of poly A and poly U tracts in the hMPV sequence spanning nt4536 to nt5623, to determine whether there is a bias between insertions of A or U. This region contains 14 poly A tracts and 3 poly U tracts with 4 or more contiguous A or U residues, respectively. Among the 15 cloned RT-PCR products amplified from the genomic RNA, 26 incidences of inserted A and 1 of inserted U were observed (Figure 3C). Thus, the data suggest a strong bias for insertions of A.

We also looked for insertions outside of the region that encoded the non-essential genes M2-1 and SH. RT-PCR was performed on rhMPV/ΔM2-2 and rhMPV total RNA to amplify the N/P, P/M, F/M2, SH/G and G/L non-coding sequences. There was a total of 23 poly A tracts and 2 poly U tracts with 4 or more contiguous A or U residues, respectively, among these sequences. However, no insertions of A were observed in the any of these non-coding sequences, showing that the high frequency of A insertions was predominantly confined to the region encoding the non-essential genes M2-1 and SH.

### An assay for detecting low frequency nucleotide insertion in rhMPV/ΔM2-2 using GFP marked viruses

**Figure 4:**
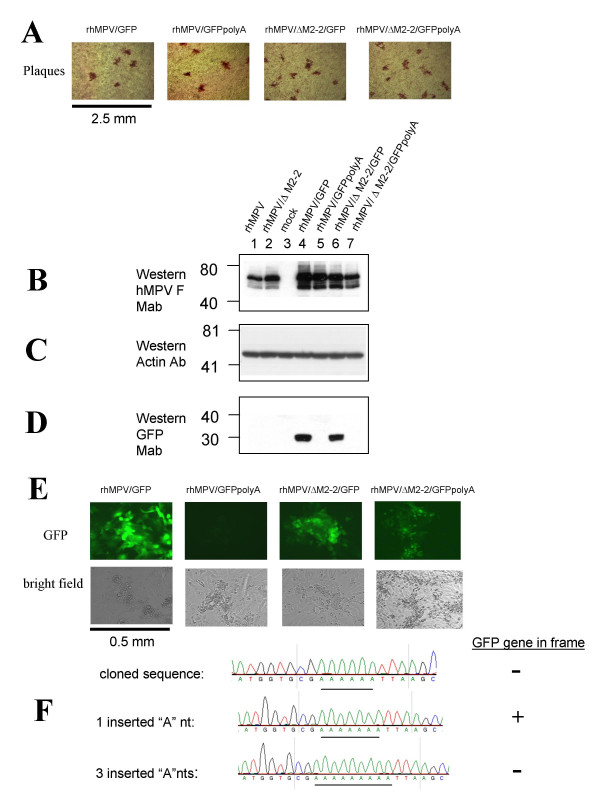
**Functional GFP expression in rhMPV/ΔM2-2/GFP6 poly A by A nucleotide insertion**. A) rhMPV and rhMPV/ΔM2-2 viruses containing the native GFP ORF or GFPpolyA sequences, harboring an engineered poly A tract that silenced the translation of GFP, formed comparable plaques in Vero cells. B) Western blots indicated F expression was comparable between viruses. C) A duplicate Western blot was probed with antibody directed to actin to serve as a loading control. D) GFP was detected by Western blot in viruses that contained native GFP ORFs. E) Fluorescence was robustly detected in viruses that contained native GFP ORFs, was readily detectable in some fluorescent foci in rhMPV/ΔM2-2/GFPpolyA, and was not detected in rhMPV/GFPpolyA. F) Nucleotide insertion of one A restored function of GFPpolyA ORF. Nucleotide insertion of 3 As would not restore functional GFPpolyA, but indicated heterogeneity at this polyA locus.

To investigate whether these insertions also occur in non-hMPV sequences, a GFP gene was inserted into the sixth gene position between the M2 and SH transcription units of rhMPV and rhMPV/ΔM2-2. An assay was developed to detect insertions, by designing a GFP ORF with an 11-nt sequence, CGA_6_TTA, positioned downstream of the first two GFP codons. This resulted in a frame shift in the downstream reading frame and a premature translational stop codon at the 6^th ^GFP codon, abrogating expression of GFP (Figure 1B,C and 1D). The modified GFP ORF is labeled GFPpolyA (Figure 1D). Insertion of a single nucleotide (or 4, 7, 10, etc.) in the A_6 _tract of the CGA_6_TTA sequence would restore the translationally silenced GFP ORF, resulting in a fluorescent hMPV infectious focus. Four full-length cDNA's were engineered to recover rhMPV/GFP, rhMPV/GFPpolyA, rhMPV/ΔM2-2/GFP, and rhMPV/ΔM2-2/GFPpolyA viruses. Titers ranged from 6.6 log_10 _PFU/mL for rhMPV/ΔM2-2/GFP to 7.3 log_10 _PFU/ml for rhMPV/GFPpolyA and plaque sizes between all four viruses were similar (Figure 4A). However, rhMPV/ΔM2-2 and rhMPV/GFP plaques were both smaller than rhMPV plaques.

Vero cells were inoculated at MOI of 0.1 with rhMPV/GFP, rhMPV/GFPpolyA, rhMPV/ΔM2-2/GFP or rhMPV/ΔM2-2/GFPpolyA as well as the control viruses rhMPV and rhMPV/ΔM2-2. Viruses were harvested on day 4 for Western blot analysis. The Western blot was probed for expression of hMPV F and GFP. Actin was also probed as a loading control (Figure 4C). The levels of hMPV F as detected by Western blot were considered equivalent among the GFP-viruses (Figure 4B). As expected GFP protein was detected by Western blot only in rhMPV/GFP and rhMPV/ΔM2-2/GFP, and not in rhMPV/GFPpolyA and rhMPV/ΔM2-2/GFPpolyA (Figure 4D). These data indicate that insertion of the GFP cassette at this genome position was well tolerated by hMPV *in vitro *and insertion of the CGA_6_TTA sequences in the N terminus of the GFP ORF effectively silenced GFP expression.

To indirectly monitor A nucleotide insertions in GFPpolyA, Vero cells were inoculated with rhMPV/ΔM2-2/GFPpolyA or one of the control viruses rhMPV/GFP, rhMPV/GFPpolyA or rhMPV/ΔM2-2/GFP, at MOI of 0.1, and viewed by fluorescence microscopy for 6 days. Fluorescence was readily observed throughout the monolayers of Vero cells infected with rhMPV/GFP or rhMPV/ΔM2-2/GFP, but not in cells infected with rhMPV/GFPpolyA (Figure 4E). Initially, no fluorescence was observed in cells infected with rhMPV/ΔM2-2/GFPpolyA. However, after two days, a few foci of fluorescent cells were observed in monolayers infected with rhMPV/ΔM2-2/GFPpolyA, suggesting that some cells were infected with GFP-expressing hMPV. One focus containing approximately a hundred infected fluorescent cells is shown (Figure 4E). The expression of GFP indicated that the reading frame of the GFP gene had been restored in some virions, and cell-to-cell spread within the focus of infection suggested that the restored GFP gene sequences were present in progeny virions. The low level of GFP expressed was only observable by fluorescence microscopy and not by Western blotting (Figure 4D).

To assess the frequency of insertions that restored expression of GFP, Vero cells in 96-well plates were inoculated with P2 stocks of rhMPV/ΔM2-2/GFPpolyA or rhMPV/GFPpoly A. GFP expression was monitored by fluorescence microscopy 4 days post infection. Plates were inoculated with 1, 10, 100 or 1000 PFU/well (Table 1). No GFP-expressing foci were observed in wells inoculated with either 100 or 1000 PFU/well of rhMPV/GFPpoly A (Table 1). In contrast, cells inoculated with 10, 100, or 1000 PFU/well of rhMPV/ΔM2-2/GFPpoly A developed fluorescent foci. Fluorescent multicellular foci were observed in 25 out of 384 wells (6%) inoculated with 10 PFU/well of rhMPV/ΔM2-2/GFPpolyA (Table 1). At 100 PFU/well of rhMPV/ΔM2-2/GFPpolyA, fluorescence was observed in 65% of the infected wells (Table 1). Finally, fluorescent multicellular foci were observed in 100% of wells inoculated with 1000 PFU/well of rhMPV/ΔM2-2/GFPpolyA. Thus, this assay shows that at least one insertion occurs out of approximately every 17 infections at 10 PFU/infection and the frequency of insertions was significantly elevated in the absence of M2-2.

**Table 1:** Frequency of GFP fluorescence in Vero cells infected with rhMPVs containing GFPpolyA insert.

Inoculum (PFU/per well):	1	10	100	1000
MOI (PFU/cell):	0.0001	0.001	0.01	0.1
	Positive*/total wells	Positive*/total wells	Positive*/total wells	Positive*/total wells
rhMPV/GFPpolyA, P2	ND	ND	0/96	0/288
rhMPV/ΔM2-2/GFPpolyA, P2	0/96	25/384	124/192	384/384

* A well was scored as positive if GFP fluorescence was observed 4 days p.i.

Viruses from 24 of the wells that exhibited fluorescence and that had been inoculated at a MOI of 0.1 were passaged once in Vero cells and each of the 24 viruses retained GFP expression. Total RNA was extracted from a mixture of cells plus supernatant and RT-PCR was performed to amplify a 1.5 kb fragment encompassing the GFPpoly A gene. The RT-PCR product was cloned into pCR2.1 and 8 individual clones were sequenced. 4 cloned GFP fragments contained the 11-nt CGA_6_TTA insert as constructed, 3 contained 1 inserted A that restored the reading frame of GFP, and 1 contained 3 inserted A nucleotides in the A_6 _tract (Figure 4F). Thus, insertions of A nucleotides occurred frequently in non-hMPV sequences as well during rhMPV/ΔM2-2/GFPpolyA replication, suggesting that misincorporation of A nucleotides is not hMPV sequence-specific.

### Up-regulation of mRNA and increased read-through at the M2 gene-end sequences in rhMPV/ΔM2-2 infected cells

**Figure 5:**
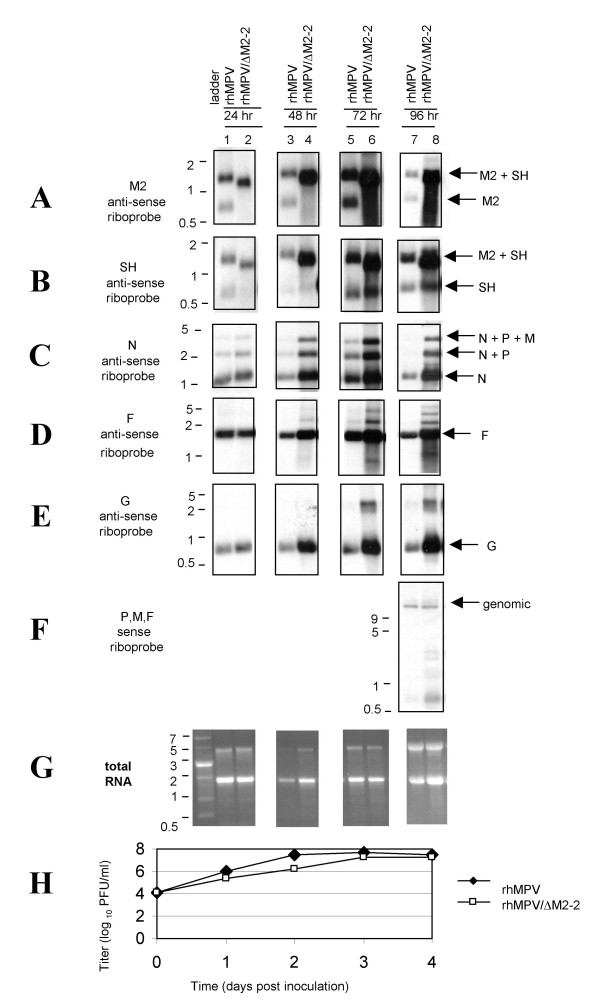
**4-day time course of Northern blot analysis and multicycle growth**. Replicate cultures of Vero cells were infected with rhMPV or rhMPV/ΔM2-2 at MOI of 0.1 PFU/cell. Cells and supernatants were harvested daily. Total RNA was extracted, and 7 replicate aliquots were separated on 1% agarose gel in the presence of 0.44 M formaldehyde gel, transferred to a nylon membrane and hybridized with digoxigenin-labeled single-stranded anti-sense riboprobes to detect mRNA as follows: A) M2 riboprobe; B) SH riboprobe; C) N riboprobe; D) F riboprobe; E) G riboprobe. F) Sense P, M, and F riboprobes were combined to detect genomic RNA. G) RNA in a duplicate gel was visualized with ethidium bromide and photographed under UV light. H) Titers of samples prior to RNA extraction were determined by plaque assay in Vero cells.

To further investigate the role of hMPV M2-2, we compared the transcription and genome replication of rhMPV/ΔM2-2 with rhMPV in Vero cells. First, we compared the amounts of rhMPV/ΔM2-2 viral transcripts with that of rhMPV by Northern blotting. Northern blot analysis was performed using hMPV-specific anti-sense DIG-labeled riboprobes to detect M2, SH, N, F, or G mRNA. At 24-hr intervals, RNA was extracted from Vero cells inoculated with rhMPV or rhMPV/ΔM2-2 at an MOI of 0.1, and Northern blot analysis was performed in 6 replicates. The M2 and SH riboprobes each detected two RNA species from rhMPV-infected cells (Lanes 1, 3, 5 and 7 of Figure 5A and 5B). The size of the minor species is consistent with the monocistronic transcript while the size of the major species coincided with the predicted size of the M2/SH read-through product. No monocistronic M2 transcripts were observed at 24 or 48 hours post rhMPV/ΔM2-2 infection in Vero cells. The predicted M2/SH read-through product showed a reduction in size in rhMPV/ΔM2-2 infected cells consistent with the deletion of M2-2 (compare lanes 1 and 2 of Figure 5A). The levels of bicistronic compared to monocistronic SH transcripts were higher in both rhMPV and rhMVP/ΔM2-2 infected cells, but the difference was more pronounced in rhMPV/ΔM2-2 infected cells (Figure 5B). This increased level of read-through was unexpected since we had sought to preserve the native M2/SH noncoding sequences. One explanation could be that transcription termination at the M2 gene end sequences required nucleotides in the coding region of M2-2 that had been inadvertently removed and/or the M2 termination signal was altered by the introduction of the Swa I site.

Next we compared the amounts of M2 transcripts in cells infected with rhMPV or rhMPV/ΔM2-2 at days 1 to 4 post-infection (p.i.). At day 1 p.i., the levels of transcripts were equivalent between both viruses (lanes 1 and 2 in Figure 5A). By day 2 p.i., the relative levels had changed markedly. The amount of transcripts in cells infected with rhMPV/ΔM2-2 was several-fold higher compared to cells infected with rhMPV (lanes 3 and 4 in Figure 5A). The up-regulation was maintained up to day 4, when peak titers were observed (lanes 7 and 8 in Figure 5). More SH, N, F, and G transcripts were also observed in cells infected with rhMPV/ΔM2-2 compared to rhMPV (Figure 5B,C,D and 5E). Therefore, M2-2 deletion resulted in up-regulation of viral transcripts of genes upstream (N, F) and downstream (SH, G) of the M2 gene. However, the increased levels of viral transcripts produced by the rhMPV/ΔM2-2 mutant were not accompanied by an increase in virus titer. On days 2 and 3, rhMPV/ΔM2-2 had higher levels of transcripts but equivalent or lower titers compared to rhMPV (Figure 5H). Neither was there a concomitant increase in protein expression, at least for the F gene (Figure 4B, lanes 1 and 2). Thus, the higher levels of viral transcripts produced by the M2-2 deletion mutant did not yield a greater number of infectious rhMPV/ΔM2-2 virions compared to rhMPV. We noted that the levels of rhMPV transcripts peaked at day 3 (lanes 1, 3, 5 and 7 in Figure 5), while the levels of rhMPV/ΔM2-2 transcripts remained the same on days 3 and 4 (lanes 6 and 8 of Figure 5).

RNA samples from day 4 were also probed for genomic (anti-sense) RNA using a mixture of three riboprobes directed to P, M and F genes. No significant differences were observed between the amount of genomic RNA in cells infected with rhMPV/ΔM2-2 and rhMPV (lanes 7 and 8, Figure 5F). Thus, deletion of M2-2 altered the ratio between hMPV genomic RNA and mRNA.

### rhMVP/ΔM2-2 is attenuated in hamsters

Syrian Golden hamsters are highly permissive for hMPV replication and were used to assess the attenuation of rhMPV/ΔM2-2 [14,18]. Groups of 8 hamsters were inoculated on day 0 with 10^6 ^PFU of *wt*hMPV/NL/1/00, rhMPV or rhMPV/ΔM2-2. Both the recombinant viruses were P3 stocks. On day 4, titers of virus in the nasal turbinates and lungs were compared. As expected, the titers of *wt*hMPV/NL/1/00 and rhMPV in nasal turbinates and lungs were comparable (Table 2). However, the titers of rhMPV/ΔM2-2 were 3.7 log_10 _PFU/gm lower in the URT and 1.8 log_10 _PFU/gm lower in the LRT, relative to rhMPV titers. Therefore, rhMPV/ΔM2-2 was approximately 10,000-fold and 100-fold more restricted in the URT and LRT, respectively, compared to rhMPV.

**Table 2:** Titers of hMPV in hamsters after immunization and after challenge.

Immunizing Virus^a^	Mean virus titer post immunization^b ^(log_10 _PFU/gm tissue +/- SE)		Mean virus titer post challenge^c ^(log_10 _PFU/gm tissue +/- SE)	
	NT	Lungs	NT	Lungs

wt hMPV/NL/1/00	5.9 +/- 0.3	4.6 +/- 1.4	<0.4 +/- 0.1	<0.4 +/- 0.1
rhMPV	6.0 +/- 0.6	5.1 +/- 0.5	<0.4 +/- 0.1	<0.4 +/- 0.1
rhMPV/ΔM2-2	2.3 +/- 0.6	3.3 +/- 0.4	<0.4 +/- 0.1	<0.4 +/- 0.1
placebo	ND	ND	5.6 +/- 0.6	4.5 +/- 1.5

^a ^Syrian golden hamsters, in groups of 8, were infected intranasally with 10^6 ^PFU/animal of the immunizing virus or placebo.

^b ^4 animals per group were sacrificed on day 4 p.i.. Nasal turbinates and lungs were harvested and virus titers were determined by plaque assay.

^c ^28 days posit immunization, 4 animals per group were challenged with 10^6 ^PFU/animal of wt hMPV/NL/1/00. 4 days post challenge, the animals were sacrificed. Nasal turbinates and lungs were harvested and virus titers were determined by plaque assay.

To determine if the lower level of replication in lungs and nasal turbinates of hamsters was sufficient to protect the hamsters from subsequent infection with hMPV, 4 hamsters were challenged with 10^6 ^PFU of *wt*hMPV/NL/1/00 4 weeks post immunization. Four days post administration of the challenge, no virus was detected in either lungs or nasal turbinates of the immunized hamsters while unvaccinated animals had 5.6 +/- 0.6 PFU/gm in URT and 4.5 +/- 1.5 PFU/gm in the LRT (Table 2). Therefore, replication of rhMPV/ΔM2-2 was restricted in hamsters and animals were protected from challenge with *wt*hMPV/NL/1/00.

## Discussion

Using reverse genetics, we engineered rhMPV lacking the M2-2 gene with the aim of generating a potential vaccine candidate. rhMPV/ΔM2-2 grew to high titer in Vero cells, was attenuated in the respiratory tract of hamsters, and protected immunized hamsters from challenge with *wt*hMPV/NL/1/00. These results agree with a similar study reported by Buchholz *et al*. in which a different subtype A hMPV strain, CAN97-83, with a deletion of M2-2 was proposed as a potential vaccine candidate [14,15]. Our studies utilized the rhMPV/NL/1/00/E93K/S101P backbone which contained engineered mutations in the hMPV F gene that allows this virus to replicate efficiently in Vero cells without trypsin supplementation [17]. This property is expected to facilitate the testing and manufacture of potential live hMPV vaccine candidates.

To assess the genetic stability of our M2-2 deletion mutant, sequence analyses were performed on P4 stocks of rhMPV/ΔM2-2. These analyses revealed major subpopulations (as high as 50%) that contained insertions of A nucleotides (sense direction) in the M2-1 and SH ORFs. These insertions appeared predominantly in A tracts and were also observed in non-hMPV sequences. Nucleotide insertions were also readily detected in an A tract introduced in the GFP ORF. Interestingly, insertions of A were not observed outside the region encompassing the non-essential genes M2 and SH. Transcriptional editing, whereby alternative reading frames of viral genes are accessed, has been observed in the P gene of several paramyxoviruses [19-23]. Therefore it is possible that an inserted A could occur frequently during transcriptional editing of paramyxovirus RNA. The nucleotide insertions observed in rhMPV M2-2 deletion mutants differ somewhat from transcriptional editing in that (i) the positions of inserted A nucleotides did not appear to be sequence biased beyond selecting for A tracts and is not hMPV sequence specific, and (ii) the nucleotide insertions were incorporated into the viral genome and could be propagated, as shown by passaging of fluorescent rhMPV/ΔM2-2/GFPpolyA viruses. Interestingly, these insertions did not appear to confer growth advantages in Vero cells because further passaging of rhMPV/ΔM2-2 promoted new A insertions and did not increase the subpopulations of earlier insertions. Many of the A nucleotide insertions caused premature translation terminations in the non-essential M2-1 and/or SH ORFs. These observations argue mechanistically against transcriptional editing and suggest that the insertions observed when M2-2 was deleted may be caused by an alteration in the fidelity of the replication complex directly or indirectly.

Removal of the hMPV M2-2 gene resulted in up-regulation of viral transcription, although there was no alteration in the level of genomic RNA. This had been observed previously for the respiratory syncytial virus (RSV) M2-2 gene as well as for hMPV [14]. Deletion of RSV M2-2 resulted in higher levels of viral transcripts compared to wt RSV. Based on these observations it was postulated that the RSV M2-2 is involved in regulating the balance between transcription and genome replication [24,25]. Our observation that the levels of rhMPV transcripts peaked at day 3 p.i., while the levels of rhMPV/ΔM2-2 transcripts remained high through day 4 p.i. is also consistent with a higher level of viral transcripts in rhMPV/ΔM2-2 infected cells. Thus, deletion of the hMPV M2-2, like its RSV counterpart, appears to cause aberrant regulation of viral transcription.

Comparison of monocistronic and polycistronic viral transcripts showed differences in the frequency of readthrough transcription at the M2 gene end sequences between rhMPV and rhMPV/ΔM2-2 infected cells. In RNA from cells infected with rhMPV, the M2 riboprobes detected a minor monocistronic M2 transcript and a major polycistronic M2/SH readthrough transcript. While transcription readthrough is not unique to the M2/SH intergenic region, the polycistronic readthrough transcripts at other noncoding regions such as N/P and F/M2 were less pronounced and monocistronic transcripts predominated. The genes immediately upstream and downstream of the M2 and SH transcription units also existed predominantly as monocistronic transcripts indicating that the M2 gene-end sequences are particularly prone to high frequency of readthrough transcription. The frequency of readthrough transcription at the M2 gene stop sequences appeared to be accentuated by the removal of the M2-2 ORF. This may in part be attributed to the sequences that were removed and/or altered by the introduction of a Swa I site at the proximity of the M2 gene end sequences. Nonetheless, the increased frequency of readthrough at this gene junction may perturb the expression of downstream genes such as SH, G and L. In rhMPV/ΔM2-2 infected cells, there are major populations of M2-1 transcripts that contained premature termination codons introduced by the high point mutation frequency. Therefore, it is possible that M2-1 expression was significantly reduced during rhMPV/ΔM2-2 infection and this reduction in M2-1 expression may also contribute to the up-regulation of transcription and increased frequency of read-through observed.

Our results differ somewhat from that reported for the recombinant CAN97-83 strain of hMPV. Growth of recombinant rΔM2-2 CAN97-83 is trypsin-dependent and peak titer was not observed until 11 days post infection [14]. In contrast, our rhMPV/ΔM2-2 achieved peak titers at 4 days post-infection, a significant savings in production time. Interestingly, both ΔM2-2 viruses showed dramatic up-regulation of transcription at 48 hours post infection despite very different growth kinetics. No increase in the frequency of read-through transcription was observed for rΔM2-2 CAN97-83 whereas we observed increased polycistronic M2/SH transcripts in rhMPV/ΔM2-2 infected cells. This may stem from differences in the construction of the M2-2 deletion. rΔM2-2CAN97-83 had a deletion of 152 nt in the M2-2 ORF whereas our construct had a deletion of 142 nt and a SwaI site introduced adjacent to the polyA tract of the M2 gene stop sequences. However, the ratio of polycistronic M2/SH transcripts to monocistronic M2 transcripts was significantly different even between the two wild-type hMPV strains, with the Netherlands strain exhibiting a higher frequency of readthrough at the M2/SH noncoding region than the Canadian strain.

Sequence analysis of rhMPV CAN 97-83, showed that mutations do develop in SH, G, L and non-coding regions (NCR), with a particularly high frequency in the SH gene [26]. While mutations and insertions were reported for rhMPV and rhMPVΔ G of the CAN 97-83 strain, the sequence analysis did not include viruses that lacked the M2-2 gene [26]. In a separate evaluation of rΔM2-2 CAN97-83, no increase in the frequency of point mutations was reported [14]. While the M2-2 proteins of both strains are completely identical, the SH protein of the CAN97-83 strain is only 83% identical to the NL/1/00 strain. There are also 26 amino acid differences in the L gene between the two strains. Finally, although deletion of the hMPV M2-2 ORF succeeded in attenuating both hMPV strains, the CAN97-83ΔM2-2 virus was more attenuated in hamsters than rhMPV/ΔM2-2 [14]. Clearly there are differences between the CAN97-83 and hMPV/NL/1/00 strains. Further study will be required to elucidate the differences in phenotype.

## Conclusion

In summary, M2-2 plays an important role in the genetic stability of the hMPV genome. Silencing of M2-2 expression resulted in a greater frequency of hMPV subpopulations harboring insertions and point mutations. Stabilizing the sequence of the rhMPV/ΔM2-2 genome by re-engineering all poly A tracts will only be partially effective because this does not address the increased frequency of point mutations. More studies are needed to gain detailed knowledge of the hMPV polymerase complex and the role of M2-2 during hMPV replication. Ablation of the M2-2 ORF also resulted in up-regulation of viral transcription but not genomic RNA and increased the frequency of readthrough transcription at the M2/SH noncoding region. Aberrant transcription regulation and increased genetic instability could both contribute to the attenuation phenotype rhMPV/ΔM2-2. Further studies are being conducted to develop a potential live hMPV vaccine candidate.

## Methods

### Cells

Vero cells (ATCC and ≤ passage 148) were maintained in minimal essential medium (MEM) (JHR Biosciences) supplemented with 10% fetal bovine serum (FBS) (Hyclone), 2 mM L-glutamine (Gibco BRL), nonessential amino acids (Gibco BRL) and 100 Units/ml penicillin G sodium with 100 ug/ml streptomycin sulfate (Biowhittaker). BSR/T7 cells (kindly provided by Dr. K. K. Conzelmann) were maintained in Glasgow MEM (Gibco BRL) supplemented with 10% FBS, 5% tryptose phosphate broth (Sigma), nonessential amino acids, 1 mg/ml G418 (Gibco BRL) and 100 Units/ml penicillin G sodium with 100 ug/ml streptomycin sulfate and 1 mg/ml G418 (Gibco BRL) every other passage.

### Viruses

HMPV viruses were propagated in Vero cells with optiMEM (Gibco BRL) containing 100 Units/ml penicillin G sodium with 100 ug/ml streptomycin sulfate. Virus stocks were harvested by scraping the cells and supernatant together with 10× SPG (10× SPG is 2.18 M sucrose, 0.038 M KH_2_PO_4_, 0.072 M K_2_HPO_4_, 0.054 M L-Glutamate at pH 7.1) to a final concentration of 1× SPG and frozen at -70°C. The virus isolate *wt*hMPV/NL/1/00 and the recombinant virus rhMPV/NL/1/00/E93K/S101P have been described previously [2,17,27]. The following recombinant viruses were generated by reverse genetics from full-length cDNA plasmids using the rhMPV/NL/1/00/E93K/S101P backbone: rhMPV/ΔM2-2, rhMPV/GFP, rhMPV/GFPpolyA, rhMPV/ΔM2-2/GFP, and rhMPV/ΔM2-2/GFPpolyA.

### Construction of full-length hMPV cDNA plasmids

The cDNA for rhMPV/NL/1/00/E93K/S101P was constructed as previously described [17]. To generate rhMPV/ΔM2-2 cDNA, two Swa I restriction sites were introduced into the SphI/ClaI subclone, which contained the SphI (nt 100) to Cla I (nt8678) fragment derived from rhMPV/NL/1/00/E93K/S101P, using a Quik change mutagenesis kit (Stratagene). The first SwaI site was positioned at nt5326, down stream of the M2-1 stop codon, and was generated with the primer 5' CAG**TGA**GCATGGTCCAATTTAAATTACTATAGAGG and its complement. The stop codon of M2-1 is in bold and the Swa I restriction site is underlined. The second SwaI site was positioned at nt5468, upstream of the native stop codon of M2-2, and was generated using the primer 5' CATAGAAATTATATATGTCAAGGCTTATTTAAATTAG and its complement. Digestion with Swa I resulted in the removal of 142 nts of the M2-2 gene. The Stu I (nt4495) to Cla I (nt8693) fragment in the subclone, containing M2-1, SH, and G genes, was transferred into the full-length rhMPV/NL/1/00/E93K/S101P cDNA to form rhMPV/ΔM2-2 as depicted in Figure 1A. rhMPV/ΔM2-2 cDNA plasmid used to recover virus was sequenced from nt4495 to nt8693 to confirm that no unexpected changes were generated by PCR during cloning.

To construct the cDNA for rhMPV/GFP, a NheI-N/P-GFP-NheI cassette was constructed with the N/P noncoding region (NCR) upstream of the GFP gene. An NheI site was introduced into the hMPV SphI/ClaI subclone at nt 5474, located at the stop codon of M2-2, using the primer 5'GCTTACTTAAGCTAGCTAAAAACACATCAGAGTGG (NheI site underlined) and its complement. Following NheI digestion, the NheI-N/P-GFP-NheI cassette was ligated into the subclone. A StuI (nt4495) to ClaI (nt8693) fragment, containing M2, GFP, SH and G genes, was isolated from the hMPV SphI/ClaI subclone and inserted into the full-length cDNA to form rhMPV/GFP.

To construct rhMPV/GFPpolyA, an 11 nt Poly A insert was cloned into the NheI-N/P-GFP-NheI cassette using the primer 5' TGAGCTAGCTTAAAAAAGTGGG**ACAAGTCAAA***ATGGTG*CGAAAAAATTA*AGCAAGGGCGAGG *(hMPV P gene start sequences is in bold, the GFP sequences are italicized, and the 11 nt poly A insert is underlined) to generate the cassette NheI-N/P-GFP-PolyA-NheI. The NheI-N/P-GFP-PolyA-NheI cassette was ligated into the hMPV SphI/Cla I subclone at the Nhe I site located at nt5474. Again the StuI (nt4495) to ClaI (nt8693) fragment of the hMPV SphI/Cla I subclone was inserted into the full-length cDNA to generate rhMPV/GFPpolyA (Figure 1B).

To construct rhMPV/ΔM2-2/GFP and rhMPV/ΔM2-2/GFPpolyA, an Nhe I site was introduced into the SwaIΔM2-2 subclone at nt5316, using the primer 5'GCACTAATCAAGTGCAG**TGA**GCTAGCATTTAAATTAG and its complement (the stop codon of M2-1 is in bold and the Nhe I site is underlined). The NheI-digested GFP-containing NheI-N/P-GFP-NheI or NheI-N/P-GFP-PolyA-NheI cassette was inserted at nt5316 to generate rhMPV/ΔM2-2/GFP or rhMPV/ΔM2-2/GFPpolyA respectively (Figure 1C).

### Generation of recombinant hMPV viruses from cDNA

Recombinant viruses were generated from cDNA as described previously [27]. Briefly, 1.2 ug of pCITE hMPV N, 1.2 ug of pCITE hMPV P, 0.9 ug of pCITE hMPV M2, 0.6 ug pCITE hMPV L, and 5 ug of full-length hMPV cDNA plasmid in 500 uL optiMEM containing 10 uL lipofectamine 2000 (Invitrogen), was applied to a monolayer of 10^6 ^BSR/T7 cells. The medium was replaced with fresh optiMEM 15 hr post transfection and incubated at 35°C for 2 to 3 days. Cells and supernatant from the transfection were harvested together and used to infect Vero cells. Virus recovery was verified by positive immunostaining 6 days post inoculation with ferret polyclonal Ab directed to hMPV. Recovered viruses were further amplified in Vero cells by inoculating at a multiplicity of infection (MOI) of 0.1 PFU/cell.

### hMPV Plaque Assay

Virus titers (plaque forming units (PFU)/ml) were determined by plaque assay in Vero cells. Monolayers of Vero cells in TC6-well plates were inoculated with 10-fold serial dilutions of virus. After 1 hour adsorption, the inoculum was aspirated, the monolayer was overlaid with optiMEM diluted 1:1 with 2% methylcellulose, and the plates were incubated for 7 days at 35°C. Plaques were immunostained with ferret polyclonal antisera directed to hMPV diluted 1:500 in PBS containing 5% powdered milk (w/v) (PBS-milk). The cells were then incubated with horseradish peroxidase-conjugated goat anti-ferret antibody (Dako) diluted 1:1000, followed by incubation with 3-amino-9-ethylcarbazole (AEC) (Dako) to visualize plaques. Ferret polyclonal antisera were generated by collecting blood 4 weeks after infection of 8–10 week old ferrets with 6.0 log_10 _*wt*hMPV/NL/1/00 administered intranasally.

### Growth of rhMPV viruses in Vero cells

Subconfluent monolayers of Vero cells in TC6-well plates were inoculated at a MOI of 0.1 PFU/cell with rhMPV or rhMPV/ΔM2-2 diluted in optiMEM. After 1 hr adsorption at 35°C, the virus inoculum was replaced with 2 ml optiMEM. Combined cells and supernatant were collected at 24 hr intervals for 4 or 6 days, stabilized with 1× SPG and frozen at -70°C. Virus titers were determined by plaque assay in Vero cells.

### Replication of rhMPV, rhMPV/ΔM2-2, and wthMPV/NL/1/00 in Syrian golden hamsters

Five-week-old Syrian golden hamsters (8 animals per group) were infected intranasally with 10^6 ^PFU/animal of virus or placebo medium in 100 uL. Four days post infection, the nasal turbinates and lungs were harvested from 4 animals per group, homogenized and titered by plaque assay in Vero cells. On day 28 post infection, immunized hamsters were challenged with an intranasal dose of 10^6 ^PFU/animal of *wt*hMPV/NL/1/00. Four days post-challenge, the nasal turbinates and lungs were harvested and assayed for challenge virus replication by plaque assay.

### RT-PCR of recovered viruses for nucleotide sequence analysis

Total RNA was extracted from hMPV-infected cells using TRizol (Invitrogen) reagent according to the manufacturer's instructions followed by phenol/chloroform (Amresco) extraction and ethanol precipitation. RT-PCR products of total hMPV RNAs were generated using a one step Ultrasense RT-PCR kit (Invitrogen) with sense and anti-sense primers designed to generate 1 to 2 kb fragments from total RNA. Genomic RNA was amplified with a two-step Super Script III platinum RT-PCR kit (Invitrogen). To ensure that only genomic RNA was amplified in the two-step process, only the sense primer was present during the RT step, the RT was deactivated by treatment of the product at 94°C for 15 minutes, and phenol/chloroform extraction was performed on the RT product to remove any residual RT enzyme prior to the PCR reaction. Sequence analysis was performed only on DNA fragments of the expected size isolated from agarose gels using a gel extraction kit (Qiagen).

### Northern blot analysis

Vero cells were inoculated at MOI = 0.1 and incubated at 35°C for 4 days. Cells and supernatant were scraped together and total RNA was extracted using Trizol reagent (Invitrogen) followed by an additional phenol-chloroform extraction and ethanol precipitation. RNA was separated on 1% agarose gel in the presence of 0.44 M formaldehyde and transferred to a positively charged nylon membrane (Amersham Biosciences). The RNA was hybridized with gene-specific riboprobes labeled with digoxigenin using a DIG RNA labeling kit (Roche). The hybridized bands were visualized with a DIG luminescent detection kit (Roche).

### Western blot analysis

Western blot analysis was performed as described previously [17]. Briefly, in duplicate, cell lysates of hMPV-infected Vero cells were separated on a 12% polyacrylamide Tris-HCl Gel (Bio-Rad), transferred to a Hybond-P polyvinylidene difluoride membrane (Amersham Biosciences) and immunostained with either hamster Ab # 121-1017-133 (MedImmune) directed to hMPV F, mouse Ab directed to GFP (Roche Molecular Biochemicals), or mouse Ab directed to actin (Chemicon MAB #1501). Bands were visualized by incubation with horseradish peroxidase-conjugated anti-hamster Mab or anti-mouse Mab, developed with chemiluminescence substrate (Amersham Biosciences), and exposed to Biomax MR film (Kodak).

## Competing interests

The authors are employed at MedImmune, Inc.

## Authors' contributions

JS participated in design and interpretation of the experiments, carried out the molecular genetic studies, performed the sequence alignments and drafted the manuscript. JK performed the growth curves and participated in cloning and recovery of the viruses. MM and JG performed the immunization and challenge experiments in hamsters. RS and RT contributed to experimental designs of the study and writing of the manuscript. All authors read and approved of the final manuscript.
